# TNF Receptor-Associated Factor 1 is a Major Target of Soluble TWEAK

**DOI:** 10.3389/fimmu.2014.00063

**Published:** 2014-02-18

**Authors:** José Antonio Carmona Arana, Axel Seher, Manfred Neumann, Isabell Lang, Daniela Siegmund, Harald Wajant

**Affiliations:** ^1^Division of Molecular Internal Medicine, Department of Internal Medicine II, University Hospital Würzburg, Würzburg, Germany; ^2^Department of Oral and Maxillofacial Plastic Surgery, University Hospital Würzburg, Würzburg, Germany

**Keywords:** CD40, NFκB, TNF, TRAF1, TWEAK

## Abstract

Soluble tumor necrosis factor (TNF)-like weak inducer of apoptosis (TWEAK), in contrast to membrane TWEAK and TNF, is only a weak activator of the classical NFκB pathway. We observed that soluble TWEAK was regularly more potent than TNF with respect to the induction of TNF receptor-associated factor 1 (TRAF1), a NFκB-controlled signaling protein involved in the regulation of inflammatory signaling pathways. TNF-induced TRAF1 expression was efficiently blocked by inhibition of the classical NFκB pathway using the IKK2 inhibitor, TPCA1. In contrast, in some cell lines, TWEAK-induced TRAF1 production was only partly inhibited by TPCA1. The NEDD8-activating enzyme inhibitor MLN4924, however, which inhibits classical and alternative NFκB signaling, blocked TNF- and TWEAK-induced TRAF1 expression. This suggests that TRAF1 induction by soluble TWEAK is based on the cooperative activity of the two NFκB signaling pathways. We have previously shown that oligomerization of soluble TWEAK results in ligand complexes with membrane TWEAK-like activity. Oligomerization of soluble TWEAK showed no effect on the dose response of TRAF1 induction, but potentiated the ability of soluble TWEAK to trigger production of the classical NFκB-regulated cytokine IL8. Transfectants expressing soluble TWEAK and membrane TWEAK showed similar induction of TRAF1 while only the membrane TWEAK expressing cells robustly stimulated IL8 production. These data indicate that soluble TWEAK may efficiently induce a distinct subset of the membrane TWEAK-targeted genes and argue again for a crucial role of classical NFκB pathway-independent signaling in TWEAK-induced TRAF1 expression. Other TWEAK targets, which can be equally well induced by soluble and membrane TWEAK, remain to be identified and the relevance of the ability of soluble TWEAK to induce such a distinct subset of membrane TWEAK-targeted genes for TWEAK biology will have to be clarified in future studies.

## Introduction

Tumor necrosis factor (TNF)-like weak inducer of apoptosis (TWEAK) and fibroblast growth factor (FGF)-inducible molecule-14 (Fn14), alternatively termed TweakR or TNFRSF12, form a phylogenetically well conserved ligand–receptor pair of the TNF family. Fn14 is highly expressed during development and in cancer but is also strongly induced in a variety of cell types of the adult organism upon tissue damage (1, 2). Accordingly, Fn14 has been implicated in tissue repair-associated processes, such as control of proliferation and differentiation of mesenchymal progenitor cells, orchestration of immune reactions, cell migration, and angiogenesis. However, if not held in check, Fn14 activity can also have detrimental effects contributing to autoimmune-related pathologies but also to muscle atrophy, ischemic tissue damage, and fibrosis (1, 2). Typically, stimulation of Fn14 results in the activation of various proinflammatory signaling pathways such as the classical and alternative NFκB pathway and the various MAPK pathways (3, 4). Like most other members of the TNF receptor family, Fn14 activates proinflammatory signaling pathways with the help of adapter proteins of the TNF receptor-associated factor (TRAF) family, especially TRAF2 (5–7). Indeed, TRAF2 and the TRAF2-associated E3 ligases cellular inhibitor of apoptosis-1 (cIAP1) and cIAP2 are not only required by Fn14 for the activation of the classical NFκB pathway but are also crucially involved in TWEAK-induced stimulation of MAP kinases (4, 8). There is furthermore evidence that TRAF2 is also required for TWEAK/Fn14-mediated activation of Rho-GTPases by recruiting the Src homology 3 domain-containing guanine nucleotide exchange factor [SGEF; (9)]. Noteworthy, TWEAK-induced activation of Rho-GTPases appears particularly relevant for cell migration of glioblastoma cells (9–11). In general, however, the question how the various Fn14-associated signaling pathways contribute, in which cell type, to the aforementioned biological effects of TWEAK and Fn14 is poorly understood. Indeed, the complexity of Fn14 signaling is particularly surprising against the background of the simple structure of the molecule. Two major mechanisms that contribute to the plasticity and variability of TWEAK/Fn14-mediated effects are based on the capability of Fn14 to adopt different states of activity and to modulate signaling by other members of the TNF receptor family.

Like other members of the TNF ligand family, TWEAK acts as a membrane-bound trimeric protein but also in a soluble form that is released from the membrane-bound molecule by proteolytically processing (12–15). It is noteworthy that the two forms of TWEAK trigger partly different patterns of intracellular signaling events. So far investigated, membrane TWEAK or oligomerized soluble TWEAK, which mimics the activity of membrane TWEAK, efficiently activate all known Fn14-associated signaling pathways including the classical NFκB pathway and proteolytic degradation of TRAF2 (15–17). Soluble TWEAK, however, fails to trigger these responses or does it only very moderately (15). Membrane and soluble TWEAK, however, are both comparably effective with respect to activation of the alternative NFκB pathway and enhancement of TNFR1-mediated cell death (15, 17). The different activity of soluble and membrane TWEAK correlates with a different effect on complexes of TRAF2 with cIAP1 or cIAP2. Both forms of TWEAK induce efficient interaction of Fn14 with TRAF2–cIAP1/2 complexes. However, in addition to this, Fn14 also triggers transactivation of the TRAF2-associated cIAPs and thus activation of the classical NFκB pathway, TRAF2 degradation, and cIAP autodegradation when stimulated by membrane TWEAK or oligomerized soluble TWEAK. The sole recruitment of TRAF2–cIAP1/2 complexes to Fn14 is typically sufficient to reduce the cytosolic pool of TRAF2–cIAP1/2 complexes (16, 17). The cytosolic pool of TRAF2–cIAP1/2 complexes, however, is crucially involved in the constitutive inhibition of the alternative NFκB pathway and also antagonizes caspase-8 activation upon TNFR1 stimulation (2). Thus, TWEAK-induced Fn14-mediated depletion of cytosolic TRAF2–cIAP1/2 complexes, irrespective of subsequent cIAP1/2 activation, can adequately explain the similar activity of soluble and membrane TWEAK regarding activation of the alternative NFκB pathway and sensitization for TNF-induced cell death. As TRAF2–cIAP1/2 complexes are also of relevance for classical NFκB signaling and MAPK activation in response to stimulation of various members of the TNF receptor family, TRAF2–cIAP1/2 depletion is presumably also responsible for the attenuated proinflammatory activities of TNFR1, TNFR2, and CD40 that has been observed in TWEAK-primed cells (17, 18–20).

TNF receptor-associated factor 1 forms heterocomplexes with TRAF2 at the expense of the formation of TRAF2 homotrimers (21). As TRAF1 and TRAF2 can differ in their affinity for the TRAF2 binding site of a particular TRAF2-interacting TNF receptor, TRAF1–TRAF2 heteromer formation can modify TRAF2-mediated TNF receptor signaling by changing the efficacy/strength of receptor–TRAF2 interaction. Indeed, the TNF receptor family member CD40 recruits TRAF2–TRAF1 heteromers less efficient than TRAF2 homotrimers and this correlates with reduced activation of the classical NFκB pathway (22). TRAF1 is furthermore a well-established target of classical NFκB-stimulating cytokines, such as TNF and IL1. In light of a possible role of TRAF1 in the crosstalk of Fn14 with other TRAF2-interacting receptors and against the background of the different effects of soluble and membrane TWEAK on the alternative and classical NFκB pathway, we evaluated here the ability of different forms of TWEAK to induce TRAF1 expression. Unexpectedly, we found that soluble TWEAK, which poorly stimulates the classical NFκB pathway, induces strong TRAF1 expression in transformed and non-transformed cells. We furthermore obtained initial evidence that soluble TWEAK-induced TRAF1 expression contributes to the ability of the TWEAK/Fn14 system to modulate the activity of CD40 signaling.

## Results

### Soluble TWEAK is superior to TNF in TRAF1 induction

**Figure 1:**
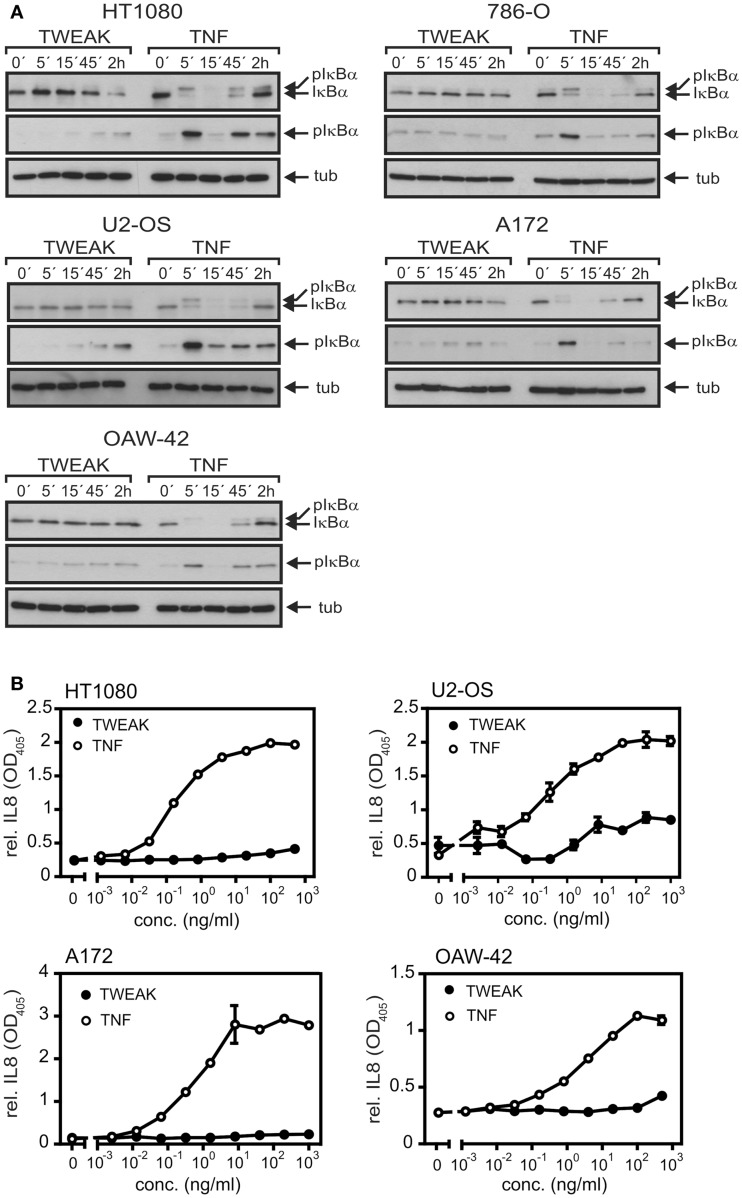
**TNF is superior to soluble TWEAK in activation of the classical NFκB pathway**. **(A)** The various cell lines were stimulated for the indicated times with Flag-TNF (100 ng/ml) and Flag-TWEAK (200 ng/ml) and total cell lysates were then analyzed for the presence of phospho-IκBα and total IκBα. **(B)**. Cells were challenged overnight in triplicates with the indicated concentrations of Flag-TNF and Flag-TWEAK and supernatants were assayed for IL8 by ELISA. 786-O cells display strong constitutive expression of IL8 and were thus not assayed for IL8 production.

The capabilities of soluble TNF and soluble TWEAK to activate the classical NFκB pathway were analyzed in various cell lines. As expected, TNF efficiently triggered phosphorylation and degradation of IκBα, two hallmarks of the classical NFκB pathway, in all cell lines investigated within 5–15 min including transformed cell lines of different origin (Figure 1A) and primary cells (Figure S1 in Supplementary Material). In contrast, in the 2-h time window investigated, soluble TWEAK showed only delayed barely detectable IκBα phosphorylation and IκBα degradation was practically not evident (Figure 1A). This furthermore correlated with the fact that production of IL8, a cytokine that is dominantly regulated by the classical NFκB pathway, was much stronger induced by TNF than by soluble TWEAK (Figure 1B; Figure S1 in Supplementary Material).

**Figure 2:**
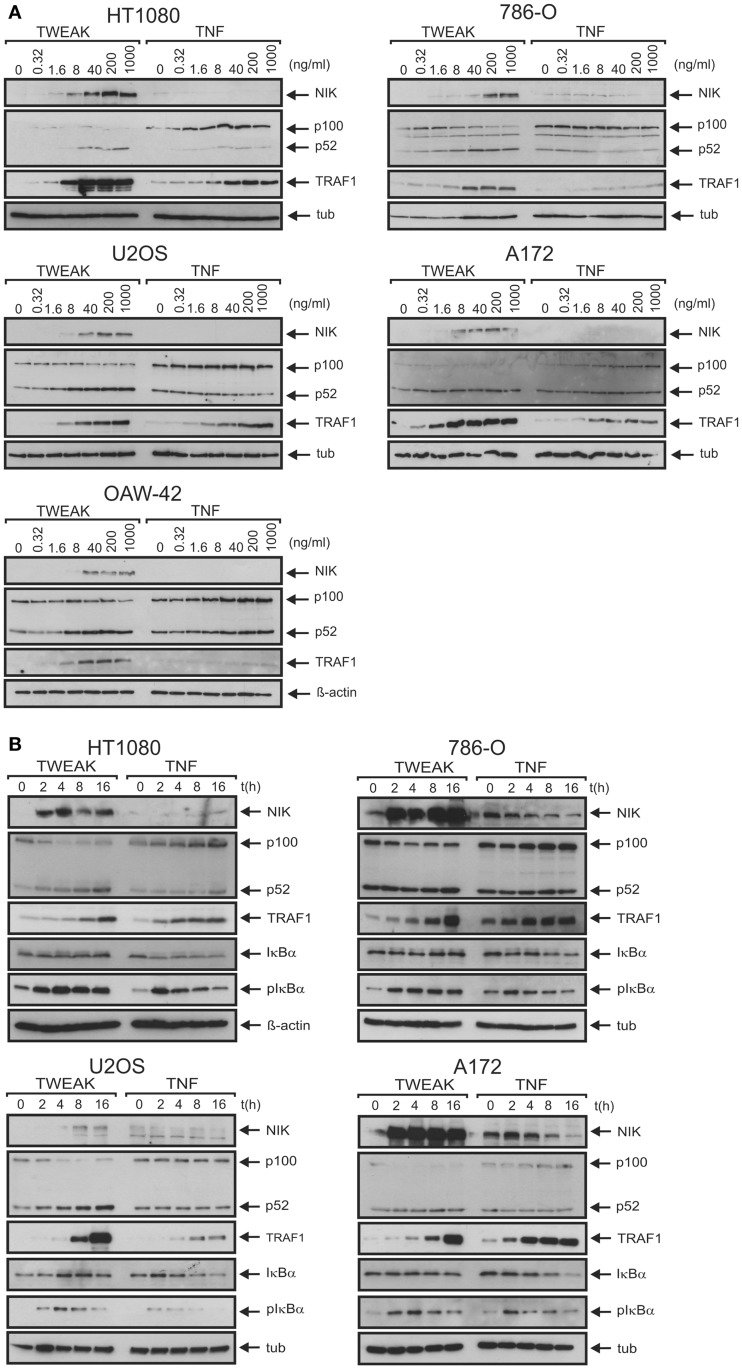
**Soluble TWEAK is superior to TNF in TRAF1 induction**. **(A,B)** Cells were either stimulated overnight with the indicated concentrations of Flag-TWEAK and Flag-TNF **(A)** or were incubated with a constant amount of these cytokines (100 ng/ml Flag-TNF, 200 ng/ml Flag-TWEAK) for varying times **(B)**. Total cell lysates were finally analyzed by western blotting with respect to the indicated proteins.

To rule out that the observed limited effects of soluble TWEAK were related to a general poor TWEAK responsiveness of the cell lines investigated or low specific activity of the soluble TWEAK batch used for stimulation, we also assayed activation of the alternative NFκB pathway. Indeed, already at low concentrations soluble TWEAK triggered accumulation of the NFκB inducing kinase (NIK) and processing of p100, two events indicative for stimulation of the alternative NFκB pathway (23), while TNF had no effect in this regard (Figures 2A,B). We also analyzed induction of TRAF1, a well-recognized NFκB-regulated target of TNF (24–26). According to the literature, there was only weak expression of TRAF1 in unstimulated cells, but TRAF1 expression was readily induced by treatment with TNF. Surprisingly, however, soluble TWEAK was as efficient as, or even superior to TNF in TRAF1 induction (Figures 2A,B; Figure S1 in Supplementary Material). Although, soluble TWEAK-induced TRAF1 expression with somewhat slower kinetics than TNF, the maximal TRAF1 levels reached were regularly significantly higher (Figures 2A,B). Noteworthy, soluble TWEAK-induced TRAF1 production occurred with some delay with respect to NIK accumulation and p100 processing. (Figures 2A,B). NIK and IKK1 can also crosstalk into the classical NFκB pathway, e.g., at the level of IκBα phosphorylation (27). We thus re-analyzed phosphorylation of IκBα in the samples of, in comparison to Figure 1A, the extended time course used for evaluation of TRAF1 induction and activation of the alternative NFκB pathway. Indeed, there was now significant phosphorylation of IκBα in soluble TWEAK-treated cells (Figure 2B). However, analysis of total IκBα levels showed no major changes in the amount of this molecule and there was only a minor fraction of the slower migrating phosphorylated IκBα species. As the gene encoding IκBα itself is a bona fide target of the classical NFκB pathway, this argues for weak but persistent stimulation of the downstream steps of the classical NFκB pathway resulting in a balance between degradation and resynthesis of IκBα. However, as soluble TWEAK, in contrast to TNF, failed to trigger robust production of IL8 (Figure 1B), this hardly explains the superior induction of TRAF1 by soluble TWEAK.

**Figure 3:**
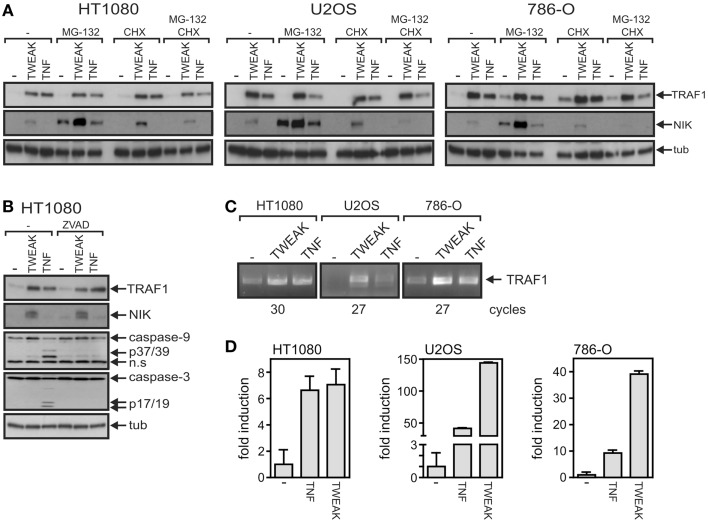
**Soluble TWEAK is superior to TNF in *TRAF1* mRNA induction**. **(A,B)** TRAF1 expression was induced in the indicated cell lines by overnight stimulation with Flag-TWEAK (200 ng/ml) or Flag-TNF (100 ng/ml). Cells were then treated for additional 4 h with the proteasome inhibitor MG132 (20 μM) and/or the protein synthesis inhibitor cycloheximide (25 μg/ml). **(B)** HT1080 cells were stimulated overnight with TNF and TWEAK in the presence and absence of z-VAD-fmk (20 μM) and total cell lysates were analyzed by western blotting for TRAF1 induction. **(C,D)** Total RNA was isolated from cells stimulated with Flag-TNF (100 ng/ml) or Flag-TWEAK (200 ng/ml) overnight and subjected to analysis by reverse transcriptase PCR **(C)** and quantitative real time PCR. The average fold induction calculated from normalized data derived of three fully independent experiments is shown **(D)**.

One possible explanation for the observation that the capabilities of TNF and soluble TWEAK to trigger the classical NFκB pathway does not correlate with the TRAF1 expression levels induced by these ligands is that in the case of TNF, the accumulation of TRAF1 is limited by post-translational mechanisms. Indeed, it has been reported that TRAF1 can be processed at D163 by caspase-3 and caspase-8 in death receptor-stimulated cells resulting in a NFκB inhibitory fragment (28–30). Thus, this mechanism could possibly not only reduce the amount of TRAF1 full-length molecules but could also terminate sustained TRAF1 induction via the classical NFκB pathway. There is furthermore evidence that cIAP2-mediated ubiquitination of TRAF1 results in proteasome-dependent degradation (31). Thus, to evaluate the possibility that differences in the post-translational regulation of TRAF1 expression between TNF- and TWEAK-treated cells are responsible for the seemingly superior TRAF1 induction by TWEAK, we analyzed TRAF1 stability in TNF- and TWEAK-stimulated cells. For this purpose, cells were treated upon TRAF1 induction with the proteasome inhibitor MG132 and the protein synthesis inhibitor cycloheximide (CHX) (Figure 3A). As TNF has a weak apoptosis-inducing effect on HT1080 cells, we investigated in this cell line also the effect of the pan-caspase inhibitor z-VAD-fmk on TRAF1 induction (Figure 3B). However, we found no evidence in these experiments that the aforementioned post-transcriptional mechanisms differentially limit TNF- and TWEAK-induced TRAF1 expression. Furthermore, more efficient TRAF1 induction by soluble TWEAK was also evident from RT-PCR analysis of TNF- and TWEAK-treated cells arguing again against a major role of post-transcriptional mechanisms in TRAF1 production by TNF and soluble TWEAK (Figures 3C,D).

### Soluble TWEAK induces TRAF1 by classical NFκB pathway-dependent and -independent mechanisms

**Figure 4:**
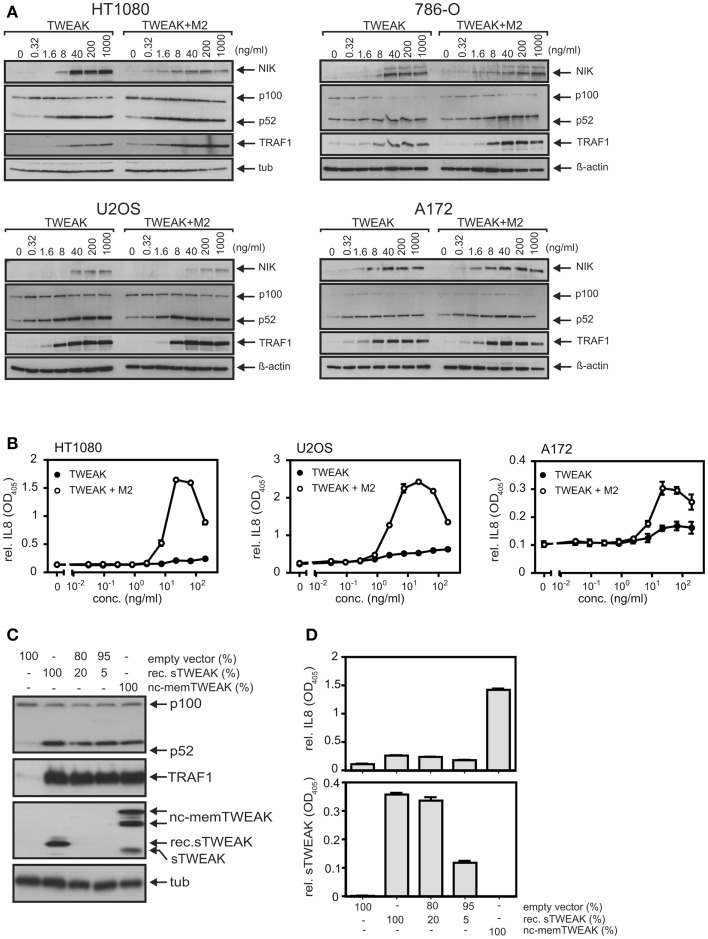
**Oligomerization of soluble TWEAK results in enhanced induction of IL8 but has no major effect on TRAF1 induction and alternative NFκB signaling**. **(A)** Cells were stimulated overnight as indicated with Flag-TWEAK and 1 μg/ml of the Flag-specific mAb M2 and TRAF1 expression in total cell lysates were analyzed by western blotting. **(B)** Cells were stimulated in triplicates with increasing concentrations of Flag-TWEAK in the presence and absence of M2 (1 μg/ml). The next day, supernatants were assayed for their IL8-content by ELISA. Prior stimulation cell culture medium was replaced to reduce the background caused by constitutive IL8 production. Please note, the decline in IL8 production observed at high concentrations of anti-Flag oligomerized Flag-TWEAK presumably reflects suboptimal complex formation by M2 and Flag-TWEAK. **(C,D)** HT1080 cells were transiently transfected with the indicated mixtures of expression plasmids encoding soluble Flag-TWEAK (rec. sTWEAK) or a non-cleavable mutant of membrane TWEAK (nc-memTWEAK) and empty vector. Next day, cells were harvested and seeded in 6-well and 96-well plates. After an additional day, total lysates derived of transfected cells (6-well) were analyzed by western blotting for expression of the indicated proteins **(C)** and supernatants (triplicates) were analyzed with respect to their IL8 and TWEAK content by ELISA **(D)**.

The superior ability of soluble TWEAK compared to TNF to induce TRAF1 as well as the kinetics of TWEAK-induced TRAF1 expression suggest that classical NFκB pathway-independent mechanisms play here a crucial role. Indeed, oligomerization of soluble TWEAK, a way to enhance the ability of soluble TWEAK to stimulate the classical NFκB pathway, which, however, has practically no effect on the stimulation of the alternative NFκB pathway (15), showed no major enhancing effect on TRAF1 induction (Figure 4A). The ability of soluble TWEAK to induce the classical NFκB target IL8, however, was strongly enhanced by oligomerization (Figure 4B). As, on the one hand, oligomerization enhances the ability of soluble TWEAK to trigger the classical NFκB pathway, and as, on the other hand, oligomerization has no effect on the dose response of TWEAK-induced TRAF1 induction, the latter seems to be controlled to a significant extent by mechanisms independent from classical NFκB signaling. In line with our previous finding that oligomerized soluble TWEAK mimics the activity of membrane TWEAK (15, 32), we furthermore observed that cells expressing a non-cleavable mutant of membrane TWEAK efficiently trigger IL8 and TRAF1 production, while soluble TWEAK producing cells showed strong TRAF1 induction but only very moderate IL8 induction (Figures 4C,D).

**Figure 5:**
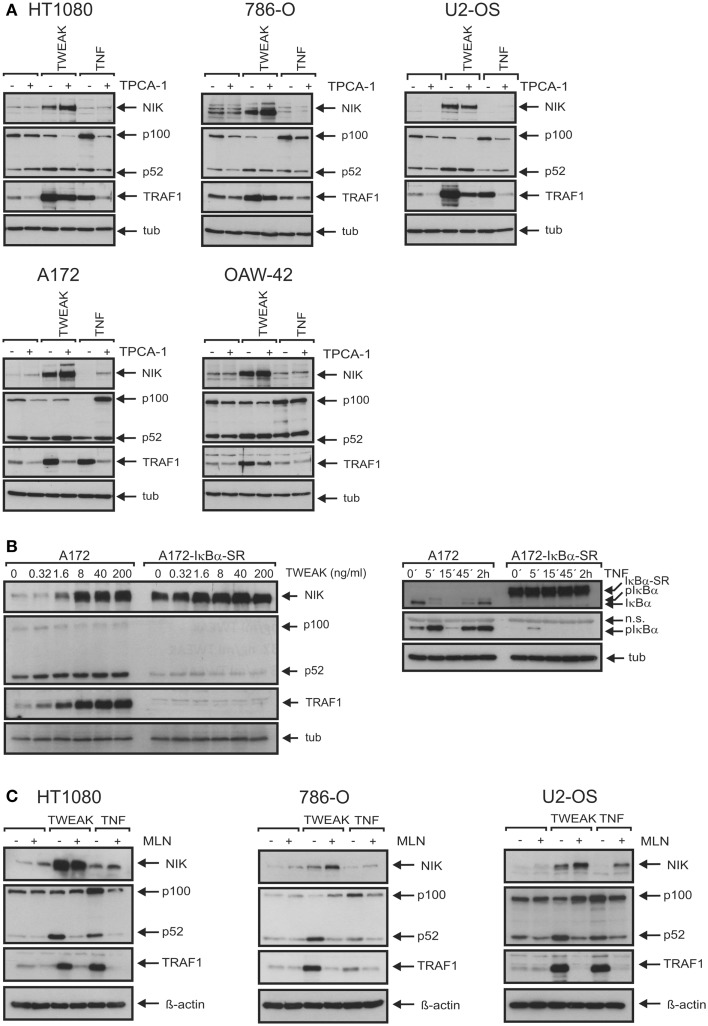
**The two NFκB signaling pathways are of varying cell type-specific relevance for TWEAK-induced TRAF1 expression**. **(A)** Cells were stimulated overnight with Flag-TWEAK (200 ng/ml) and Flag-TNF (100 ng/ml) and total cell lysates were analyzed by western blotting for NIK accumulation, p100 processing and expression of TRAF1, where indicated cells were pretreated for 30 min with 10 μM TPCA1. **(B)** Control vector and IκBα-SR transduced A172 cells were stimulated overnight with increasing concentrations of Flag-TWEAK and were assayed by western blotting of total cell extracts for TRAF1 production and activation of the alternative NFκB pathway (left panel). Efficacy of IκBα-SR-mediated repression of the classical NFκB pathway was proved by analyzing phosphorylation and degradation of IκBα in cells stimulated with TNF (100 ng/ml) (right panel). **(C)** The indicated cell lines were pretreated or not with 10 μM MLN4924 for 30 min and then stimulated with Flag-TWEAK (200 ng/ml) and Flag-TNF (100 ng/ml). The next day, total cell lysates were prepared and analyzed for the presence of the indicated proteins by western blotting.

We have recently shown that TNF-induced IKK2-mediated activation of the classical NFκB pathway is strongly inhibited without a significant effect on TWEAK-induced IKK1-mediated activation of the alternative NFκB pathway in cells treated with the IKK2-specific inhibitor TPCA1 (33). Under such conditions, TNF-induced TRAF1 production was blocked in all cell lines investigated (Figure 5A). In some cell lines, the minor levels of basal TRAF1 expression were also reduced by treatment with TPCA1. In these cases, TPCA1 treatment reduced TNF-induced TRAF1 expression to the level or even below the level of basal expression of the untreated cells (Figure 5A). This emphasizes the efficacy of the inhibitory effect of TPCA1 and indicates that basal TRAF1 expression is at least partly maintained in some cell lines by weak constitutive classical NFκB signaling. The effect of TPCA1 on TWEAK-induced TRAF1 production, however, varied dependent on the cell line considered. TWEAK-induced TRAF1 expression was fully inhibited in TPCA1-treated A172 cells and likewise in IκBα-SR expressing A172 transfectants (Figures 5A,B). However, in the other cell lines investigated there was only partial inhibition of TWEAK-induced TRAF1 expression by TPCA1 (Figure 5A). This also argues for a capability of TWEAK to induce TRAF1 by classical NFκB pathway-independent mechanisms. Moreover, in all cell lines analyzed, including those where soluble TWEAK induces significant TRAF1 expression in the presence of TPCA1, the NEDD8-activating enzyme (NAE)-inhibitor MLN4924 (34), which interferes with IκBα degradation and p100 processing (33), inhibited upregulation of TRAF1 (Figure 5C). Although, MLN4924 also targets other signaling pathways and the cell cycle, this points to an important role of the alternative NFκB pathway in soluble TWEAK-induced TRAF1 expression.

### TWEAK priming and ectopic TRAF1 expression interfere with CD40 signaling

**Figure 6:**
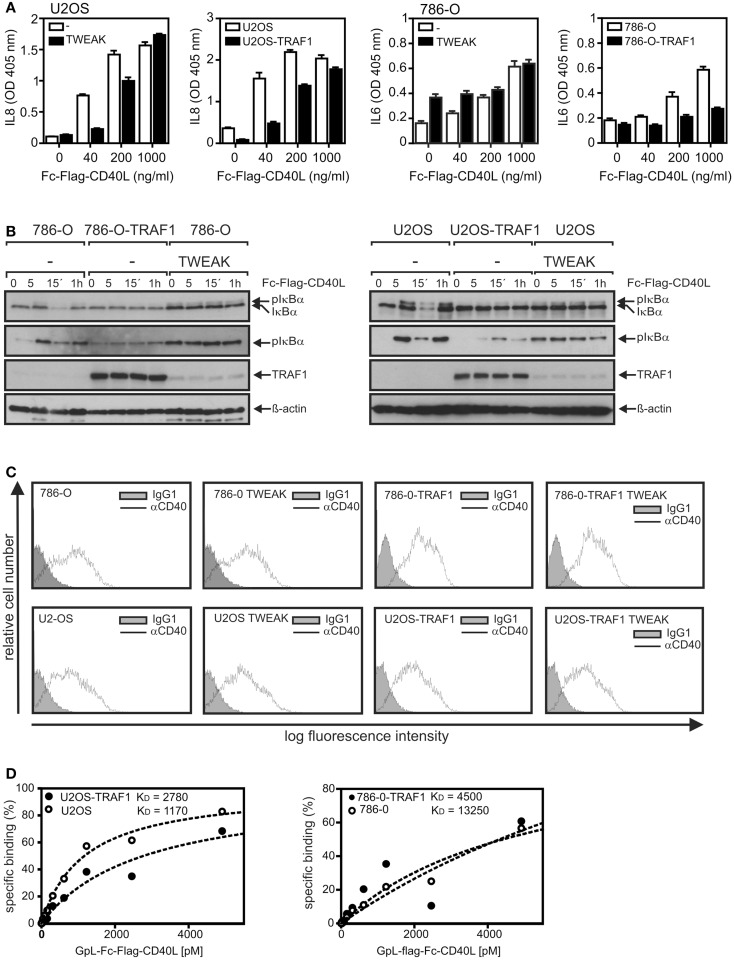
**TRAF1 expression interferes with CD40-induced signaling**. **(A)** U2OS and 786-O cells with and without TWEAK priming (200 ng/ml, 6 h) and U2OS-TRAF1 and 786-O-TRAF1 transfectants were stimulated in 96-well plates in triplicates with the indicated concentrations of Fc–Flag-CD40L. Next day, supernatants were assayed for production of IL8 or IL6 by ELISA. Prior stimulation cell culture medium was replaced to reduce the background caused by constitutive cytokine production. **(B)** U2-OS and 786-O cells with and without TWEAK priming and TRAF1 expressing U2-OS and 786-O transfectants were stimulated with Fc–Flag-CD40L for 5 and 15 min and were finally analyzed by western blotting to detect the indicated molecules. Please note, in the case of the TRAF1 western blots a relatively short exposure time is shown to ensure reasonable visibility of overexpressed and TWEAK-induced TRAF1. **(C)** 786-O and U2-OS cells and their corresponding TRAF1 transfectants were primed overnight with soluble TWEAK or remained untreated and were then analyzed by FACS for CD40 cell surface expression. **(D)** Cells (2 × 10^5^ cells/well) were seeded in 24-well plates. Next day, half of the samples of each cell type were pretreated for 1 h at 37°C with 2 μg/ml of Fc–Flag-CD40L. Next, untreated and Fc–Flag-CD40L pretreated cells were incubated pairwise with increasing concentrations of GpL–Fc–Flag-CD40L (1 h, 37°C), a fusion protein of Fc–Flag-CD40L with the luciferase from *Gaussia princeps*. After removal of unbound CD40L molecules, cells were scratched in 50 μl medium to measure cell-associated luciferase activity. Specific binding values were obtained by subtraction of the non-specific binding values derived of Fc–Flag-CD40L pretreated cells from the corresponding total binding values. Specific binding values were fitted by non-linear regression using the GraphPad Prism5 software. Specific binding values were normalized according to the maximum binding value obtained from the linear regression.

We observed recently that priming of cells with soluble TWEAK for a few hours strongly inhibits CD40 signaling and traced this back to impaired formation of TRAF2–cIAP1/2 containing CD40 signaling complexes (20). Noteworthy, TRAF1 forms with high efficiency heterotrimers with TRAF2 and this heteromeres interact much stronger with cIAP2 than homotrimeric TRAF2 (21). On the other side, however, there is evidence that TRAF1-TRAF2 heteromers bind weaker to CD40 than TRAF2 homotrimers (22). To evaluate a possible role of TRAF1 induction in the crosstalk of TWEAK and CD40, we took advantage of 786-O and U2-OS cells that we have stably transfected for another project with a caspase cleavage-resistant TRAF1 variant with unchanged protein–protein interaction properties. In accordance with our previous findings (20), priming with soluble TWEAK diminished CD40L-induced upregulation of the classical NFκB-controlled cytokine IL8 in U2-OS cells as well as induction of the likewise classical NFκB-regulated cytokine IL6 in 786-O cells (Figure 6A).

More important, analysis of the TRAF1 transfectants also revealed reduced CD40L-stimulated cytokine induction (Figure 6A). A similar inhibitory effect of TWEAK priming and ectopic TRAF1 expression was also evident from western blot analysis of CD40L-induced phosphorylation and degradation of IκBα. In accordance with the delayed weak activation of the classical NFκB pathway already observed in Figure 2B, 786-O and U2-OS cells that were primed overnight with soluble TWEAK showed increased levels of phosphorylated IκBα compared to non-primed cells (Figure 6B). More important, however, while rapid (5–15 min) CD40L-induced phosphorylation and degradation of IκBα were detectable in non-primed 786-O and U2-OS cells, CD40 stimulation failed to trigger a significant reduction in the IκBα levels in TWEAK-primed cells as well as in TRAF1 transfectants. Moreover, there was no further CD40L-induced increase in “basal” IκBα phosphorylation in the TWEAK-primed cells and no or only a minor effect on IκBα phosphorylation in the TRAF1 transfectants (Figure 6B). As already observed earlier (20), priming with soluble TWEAK showed neither a major effect on CD40 cell surface expression nor on the affinity of CD40L for CD40 (Figures 6C,D). Similarly, TRAF1 transfectants showed no disturbance of cell surface expression of CD40 and CD40L–CD40 interaction (Figures 6C,D). Thus, changes in CD40 expression cannot explain the inhibitory effect of TWEAK priming and ectopic TRAF1 expression on CD40 signaling.

## Discussion

The proteins of the NFκB family (RelA/p65, RelB, cRel, p50, and p52) form homo- and heterodimeric transcription factors whose nuclear translocation and activity are controlled by two prototypic signaling pathways: the classical (canonical) and the alternative (non-canonical) NFκB pathway (23, 35). In non-stimulated cells NFκB dimers are retained in the cytoplasm either by interaction with proteins of the IκB family or due to the activity of an inhibitory domain, which is part of the p50 and p52 precursor proteins p100 and p105. The classical NFκB pathway can be activated by various stimuli, including proinflammatory cytokines. The latter initially trigger, with the help of E3 ligases TRAF2, cIAP1, cIAP2, and TRAF6, activation of a subset of MAP3Ks, e.g., MEKK3 and TAK1. These MAP3Ks stimulate then the IκB kinase (IKK) complex, which is composed of the regulatory scaffold protein IKKγ/NEMO and the kinases IKK1 and IKK2. IKK complex-catalyzed phosphorylation of IκBs leads next to the ubiquitination and proteasomal degradation of this inhibitory proteins and thus to release and nuclear translocation of IκB-bound NFκB dimers (35). The signaling events of the classical NFκB pathway are fast and typically result in IκB degradation and NFκB nuclear translocation in <1 h. Activation of the alternative NFκB pathway is primarily stimulated by some TNF receptors and crucially bases on the accumulation of the constitutively active MAP3K NIK. In non-stimulated cells, there is ongoing proteasomal degradation of NIK which is triggered by the concerted action of the aforementioned E3 ligases cIAP1, cIAP2, and TRAF2 and the TRAF2-related E3 ligase TRAF3. Stimulation of TNF receptors that activate the alternative NFκB pathway results in depletion and/or degradation of the NIK inhibitory E3 ligases and thus in accumulation of NIK. The latter now activates IKK1 and IKK1 in turn triggers processing of p100 to p52 and nuclear translocation of the remaining p52 containing NFκB dimers (23).

NFκB-mediated induction of TRAF1 is well established for a variety of inducers of the classical NFκB pathway, including TNF, IL1, T-cell and B-cell receptor-stimulating antibodies, LPS, and phorbol-12-myristate-13-acetate (PMA) (24–26, 36). Soluble TWEAK furthermore has been recognized as poor inducer of genes regulated by the classical NFκB pathway particular in comparison with TNF [(12, 15, 32); see also Figure 1]. Against this background, it was surprising to note in this study that soluble TWEAK is superior to TNF in TRAF1 induction, although it barely induced other classical NFκB-regulated genes, such as the gene encoding IL8 (Figures 1 and 2). This suggests that soluble TWEAK utilizes classical NFκB-independent mechanisms for TRAF1 induction. So far the only Fn14-associated signaling events for which robust triggering by soluble TWEAK had been demonstrated are depletion of TRAF2–cIAP complexes along with stimulation of the alternative NFκB pathway and enhancement of TNFR1-induced apoptosis (15, 17). It is therefore tempting to speculate that the alternative NFκB pathway plays an important role in TWEAK-induced TRAF1 expression. This idea is supported by several lines of evidence. First, the kinetic of TWEAK-induced TRAF1 expression is delayed in comparison to the kinetic of TRAF1 induction by TNF and follows the activity of the alternative NFκB pathway (Figure 2). Second, oligomerization of soluble TWEAK, which converts soluble TWEAK trimers into a molecule species with membrane TWEAK-like activity and thus a strongly enhanced capacity to trigger the classical NFκB pathway, has no major effect on TRAF1 induction although it leads to potentiation of induction of IL8 (Figure 4). Third, p80HT, a lymphoma-associated truncated mutant of p100 lacking parts of the inhibitory domain of the molecule, interacts with the TRAF1 promoter and induces TRAF1 expression (37). Fourth, the IKK2-selective inhibitor TPCA1, which only blocked activation of the classical NFκB pathway, inhibited TNF-induced TRAF1 production completely but showed in most cell lines, investigated here only a partial inhibitory effect on TRAF1 expression (Figure 5A). In contrast, the NAE inhibitor MLN4924, which inhibits signaling by both NFκB pathways, completely blocked TWEAK-induced TRAF1 expression (Figure 5C). The results of the inhibitor studies indeed argue for a role of the alternative NFκB pathway in TWEAK-induced TRAF1 expression but also point to a contribution of the classical NFκB pathway. This becomes particularly evident in the A172 cell line where TWEAK-induced TRAF1 production was completely abrogated by TPCA1 treatment (Figure 5A) or IκBα-SR expression (Figure 5B) despite poor activation of the classical NFκB pathway (Figure 1). In sum, our data suggest that both the classical and the alternative NFκB pathway act together to realize the strong induction of TRAF1 by TWEAK. In this context, it is worth mentioning that various cross-talk mechanisms have been identified in the recent years that link the two NFκB signaling pathways. These mechanisms reach from NIK- and IKK1-mediated phosphorylation of IκBα (27) over phosphorylation and transactivation of c-Rel by NIK (38) to IKK1-mediated chromatin remodeling (39, 40). Moreover, the NIK/IKK1-regulated p52 precursor protein p100 can inhibit NFκB dimers intermolecularly with the result that NFκB dimers, which are actually regulated by the classical pathway, come under the control of the alternative pathway (41).

We and others have observed that priming with soluble TWEAK for a few hours can induce a state of reduced responsiveness for proinflammatory signaling by TNFR1, TNFR2, and CD40 in a variety of cell lines and cell types including adipocytes and fibroblast-like synoviocytes of patients suffering on rheumatoid arthritis [(17, 18–20); see also Figure 6]. Although, the molecular basis of the desensitizing effect of soluble TWEAK has not been intensively studied so far, there is good evidence that TWEAK-induced Fn14-mediated depletion of cytosolic complexes of TRAF2 and cIAP1/2 plays a major role by limiting the availability of these proteins for the aforementioned receptors. For example, TRAF2 and cIAPs are recruited to the TNFR1 signaling complex and are required there for ubiquitination of RIP and subsequent recruitment of the classical NFκB-stimulating IKK complex [for review, see (42)]. However, recruitment of TRAF2 and especially of the TRAF2-interacting cIAPs and the IKK complex to the TNFR1 signaling complex as well as RIP ubiquitination are severely reduced in TWEAK-primed cells (17). Reduced recruitment of cIAP1 and cIAP2 and the IKK complex along with reduced classical NFκB signaling in TWEAK-primed cells have also been observed for TNFR2 (20). Depletion of cytosolic TRAF2–cIAP1/2 complexes straightforwardly explains the desensitizing effect of soluble TWEAK on activation of the classical NFκB pathway by TNFR1, TNFR2, and CD40 because all these receptors exploit TRAF2 and the cIAPs for activation of this pathway (4, 42). In the case of CD40, however, there is evidence for an additional mechanism. Immunoprecipitation of ligand-associated receptor signaling complexes in TWEAK-primed cells resulted for TNFR1 and TNFR2 in practically unchanged amounts of co-precipitated receptor and only showed a reduction in the amount of co-precipitated TRAF2, cIAPs, and IKK molecules (17, 20). In contrast, CD40L immunoprecipitates of TWEAK-primed cells contained only minor amounts of co-immunoprecipitated CD40 despite unchanged CD40 expression and normal CD40L–CD40 interaction on intact cells (20). It is thus tempting to speculate that TRAF2 trimers “crosslink” neighboring CD40L–CD40 complexes and that this result in supramolecular CD40–CD40L clusters that counteract the dissociation of CD40–CD40L complexes in the disintegrated cell during immunoprecipitation. Indeed, previous findings showed that CD40 poorly interacts with TRAF2–TRAF1 heteromers and preferentially binds TRAF2 homotrimers whereas TNFR1 and TNFR2 recruit TRAF2–TRAF1 heteromers with high efficacy. Against the background that we observed strong induction of TRAF1 already by soluble TWEAK (Figures 1–3), it appears possible that soluble TWEAK-induced TRAF1 expression affects recruitment of TRAF2 to CD40 and the formation/stability of supramolecular CD40L–CD40 clusters. Indeed, it has been found that TRAF1 and TRAF2 preferentially form TRAF1_1_–TRAF2_2_ heterotrimers and that these TRAF1_1_–TRAF2_2_ heterotrimers have a significantly higher affinity for cIAP2 than TRAF2 homotrimers (21). The favored formation of TRAF1_1_–TRAF2_2_–cIAP complexes along with their weak affinity for CD40 might result in reduced recruitment of cIAP-containing TRAF complexes to CD40 and could further enhance the effect of Fn14-mediated depletion of TRAF2-containing complexes. As TRAF2–TRAF1 heteromers strongly interact with TNFR2 and TNFR1-associated TRADD [e.g., Ref. (43)] this mechanism would not be of relevance for the crosstalk of TWEAK with TNFR1 and TNFR2. In line with the set fourth hypothesis, we observed in this study that ectopic expression of TRAF1 has principally a similar effect on CD40 signaling as priming with soluble TWEAK. Both TWEAK priming and ectopic TRAF1 expression inhibited CD40L-induced activation of the classical NFκB pathway (Figures 6A,B) without disturbing CD40 expression (Figure 6C) or CD40L–CD40 interaction [Ref. (20) and Figure 6D].

## Materials and Methods

### Cell lines and reagents

The human renal cell carcinoma cell line 786-O, the human osteosarcoma cell line U2-OS, the human fibrosarcoma cell line HT1080 were from the American Tissue Culture Collection. The human ovarian cancer cell line OAW-42 were from the European Typical Culture Collection and vector and IκBα-SR transduced A172 cells were a kind gift of Prof. Simone Fulda (University of Frankfurt) that has been characterized in detail elsewhere (44). With exception of 786-O cells, all cells were cultivated in RPMI1640 medium (Sigma) supplemented with 10% fetal calf serum (Invitrogen). 786-O cells were maintained in 10% FCS supplemented DMEM (Sigma). The characterization of HT1080-CD40 transfectants as well as cloning, production, and purification of Flag-TWEAK, Flag-TNF, and Fc–Flag-CD40L have been described elsewhere (15, 45). MG132 and cycloheximide were purchased from Sigma and z-VAD-fmk from Bachem. Human skeletal muscle progenitor cells including growth medium and supplements were from PeloBiotech GmbH and hMSCs were a kind gift from Dr. Joachim Nickel (University Hospital of Würzburg).

### Transfection of HT1080 cells

HT1080 cells were transfected with expression plasmids encoding Flag-tagged soluble TWEAK (Flag-sTWEAK) or non-cleavable full-length TWEAK (nc-memTWEAK) harboring mutations (R80A–R93A–R97A) that strongly reduce processing by furin proteases using Lipofectamine according to the suppliers protocol (Invitrogen). Next day, cells were harvested and seeded in 6-well and 96-well plates. After an additional day, cells from 6-wells were used for western blot analysis of total cell lysates and cell culture supernatants of cells cultivated in 96-well plates were subjected to ELISA to determine the content of IL8 (see below) and soluble TWEAK (ELISA Duo Set; R&D Systems).

### Equilibrium binding studies with GpL–Flag-CD40L

The interaction of CD40L with cell surface expressed CD40 was investigated by equilibrium binding studies at 37°C with a *Gaussia princeps* luciferase (GpL) fusion protein of Fc–Flag-CD40L as recently described elsewhere (20).

### Western blotting

For analysis of NIK accumulation, p100 to p52 processing, expression of TRAF1 and TWEAK, and phosphorylation and degradation of IκBα cells were washed once with PBS, harvested using a rubber policeman, centrifuged, and then directly lysed in 4× Laemmli sample buffer (approximately 1 × 10^6^ cells per 100 μl buffer; 5 min, 95°C) supplemented with complete protease inhibitor from Roche Applied Science and phosphatase inhibitor mixtures I and II from Sigma. Lysates were sonicated for 15 s with maximum amplitude (UP100H Ultrasonic Processor, Hielscher, Germany), heated for 5 min at 95°C and centrifuged for 3 min (Eppifuge, full speed) to remove residual insoluble debris. Lysates were further processed using standard protocols for SDS-PAGE and immunoblotting using horse radish peroxidase-conjugated secondary antibodies (Dako) and the ECL Western blotting detection reagents and analysis system (Amersham). Primary antibodies used were anti-IκBα (clone C35A5, Cell Signaling), anti-pIκBα (clone 14D4, Cell Signaling), anti-NIK (#4994, Cell Signaling), anti-p100/p52 (#05-361, Upstate), anti-TRAF1 (H-132, Santa Cruz), anti-TWEAK (#AF1090, R&D Systems,) and anti-tubulin (clone DM1A, Neomarker).

### RT-PCR and qPCR

Total RNA was purified using the RNeasy Mini kit from Qiagen according to the protocol of the supplier. For reverse transcription PCR, 1 μg of total RNA were used to synthesize cDNA with the QuantiTect Reverse Transcription kit (Qiagen) which were then subjected to conventional PCR using the TRAF1-specific primers 5′-GCCCCTGATGAGAATGAGTT-3′ (forward) and 5′-CCTGGTGACATTGGTGATCTT-3′ (rewind) and the following program: I. 95°C, 2 min; II. 20 s at 95°C followed by 60 s at 63°C and 45 s at 70°C; III. 5 min, 72°C. For quantitative real time PCR 2% of the reverse transcription PCR reaction obtained with 1 μg total RNA were subjected to real time PCR using the BioRad Cycler CT1000 with the CFX96 Real Time System and the QuantiTect SYBR Green PCR Kit (Qiagen). Following program was used: I. 95°C, hot start; II. 40 Cycles of 15 s at 95°C followed by 30 s at 52°C and 30 s at 72°C and a sample without cDNA served as a negative control. TRAF1 primers used for amplification were 5′-CATGAGAGGGGAGTATGATG-3′ (forward) and 5′-GAAGAAGAGTGGGCATCCAC-3′ (rewind). PCR Amplification of ß-actin using commercially available primers (Qiagen) was used for normalization. Fold-induction values for TRAF1 were calculated as follows: the CT-values of the β-actin PCR reactions of the TNF and TWEAK-treated samples were set to the CT-value of the β-actin PCR reaction of the cDNA derived of untreated cells. The differences between the latter and the CT-values of the β-actin PCR reactions of the TNF and TWEAK-treated samples were furthermore used to correct the corresponding CT-values of the TRAF1 PCR reactions. The differences between the corrected CT-values of the untreated and TNF- or TWEAK-treated samples (ΔCT) were finally transformed into fold induction (fold induction = 2^ΔCT^).

### Analysis of IL8/IL6 production

Cells were seeded with a density of 2 × 10^4^ cells/well in 96-well tissue cultures plates and cultivated for a day. To reduce the background of basal IL8 production, medium was replaced by fresh medium prior overnight stimulation with Flag-TNF or Flag-TWEAK. Supernatants were cleared by centrifugation and assayed for IL8/IL6-production by help of the BD OptEIA Human IL8 ELISA Kit or the BD OptEIA Human IL6 ELISA Kit according to the protocol of the manufacturer (BD Biosciences).

### Statistical analyses

Statistical analyses were performed with the help of the program GraphPad Prism 5 (GraphPad Software, Inc., La Jolla, CA, USA). Error bars shown in ELISA and RT-PCR experiments represent the standard error of mean (SEM) of triplicates. ELISA data shown are representative for two or more independent experiments. The comprehensive side by side analyses of the dose response and kinetics of TNF- and TWEAK-induced TRAF1 expression were performed only once for most cell lines whereby the basic findings (no enhancing effect of oligomerization of soluble TWEAK, comparable or stronger TRAF1-inducing activity of soluble TWEAK compared to TNF) were previously observed in two or more experiments with slightly varying conditions (stimulation time, concentrations used for stimulation, and the like).

## Author Contributions

José Antonio Carmona Arana and Manfred Neumann performed the western blot analysis of TNF- and TWEAK-stimulated TRAF1 induction and the ELISA experiments. Axel Seher did the qPCR analysis, Isabell Lang produced the recombinant proteins, Daniela Siegmund analyzed the stability of TRAF1, and Harald Wajant wrote the paper and supervised the project.

## Conflict of Interest Statement

The authors declare that the research was conducted in the absence of any commercial or financial relationships that could be construed as a potential conflict of interest.

## Supplementary Material

The Supplementary Material for this article can be found online at http://www.frontiersin.org/Journal/10.3389/fimmu.2014.00063/abstract
