# Supplementary material for: TNF Receptor-Associated Factor 1 is a Major Target of Soluble TWEAK
Source: Front Immunol. 2014 Feb 18;5:63. doi: 10.3389/fimmu.2014.00063 (PMC3927163; doi:10.3389/fimmu.2014.00063)
Supplement: Supplementary file 1 [file 71883_Wajant_DataSheet1.PDF]

Supplemental data

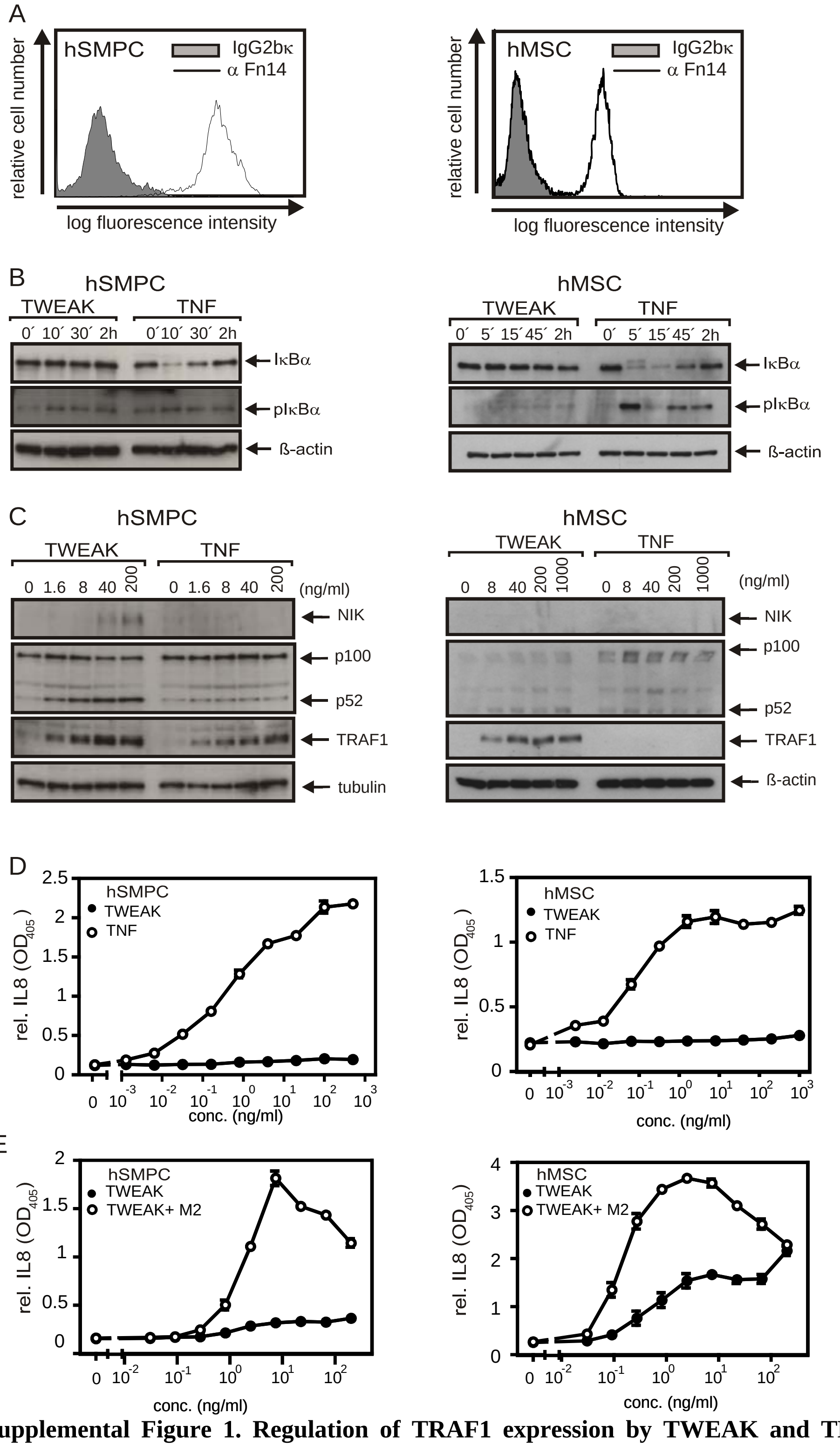

Supplemental Figure 1. Regulation of TRAF1 expression by TWEAK and TNF in primary cells. (**A**) Human skeletal muscle progenitor cells (hSMPC) and pooled mesenchymal stem cells (hMSC) were analyzed by FACS for Fn14 cell surface expression. (**B**) hMSC and hSMPC were challenged for the indicated times with Flag-TNF (100 ng/ml) and Flag-TWEAK (200 ng/ml) and total cell lysates were assayed for the presence of phospho-IκBα and total IκBα by western blotting. (**C**) Cells were stimulated overnight with the indicated concentrations of Flag-TWEAK and Flag-TNF and total cell lysates were finally analyzed by western blotting with respect to the expression of the indicated proteins. (**D**) hMSC and hSMPC were stimulated overnight in triplicates with increasing concentrations of Flag-TNF and Flag-TWEAK and secreted IL8 were finally quantified by ELISA. (**E**) Cells were stimulated in triplicates with increasing concentrations of Flag-TWEAK in the presence and absence of M2 (1 µg/ml). The next day, supernatants were again assayed for their IL8-content by ELISA. Please note, the decline in IL8 production observed at high concentrations of oligomerized Flag-TWEAK is presumably due to suboptimal complex formation by M2 and Flag-TWEAK.
